# Prevalence, correlates and pattern of Hepatitis B among antenatal clinic attenders in Yaounde-Cameroon: is perinatal transmission of HBV neglected in Cameroon?

**DOI:** 10.1186/1471-2393-13-158

**Published:** 2013-08-08

**Authors:** Nelson J Fomulu, Frederick LI Morfaw, Judith N Torimiro, Philip Nana, Mve V Koh, Takang William

**Affiliations:** 1 Department of Obstetrics and Gynaecology, Faculty of Medicines and Biomedical Sciences, University of Yaounde 1, Yaounde, Cameroon; 2 Centre Hospitalier et Universitaire de Yaounde (CHUY), Yaounde, Cameroon; 3 Centre for the Study and Control of Communicable Disease (CSCCD), Faculty of Medicines and Biomedical Sciences, University of Yaounde 1, Yaounde, Cameroon; 4 ‘Chantal Biya’ International Reference Centre for Research on HIV/AIDS Prevention and Management (CIRCB), Yaounde, Cameroon

**Keywords:** Hepatitis B, Pregnancy, Prevalence, Risk factors, Low resource setting

## Abstract

**Background:**

Few studies have evaluated the prevalence of HBV in the general Cameroonian population or among antenatal attendants. The aim of this study was to determine the prevalence, correlates and patterns of Hepatitis B surface antigen among pregnant women attending antenatal care in Yaounde-Cameroon.

**Methods:**

This was a cross-sectional multicenter study carried out in a referral hospital and two secondary hospitals in Yaounde, the capital of Cameroon. The study lasted 15 months (March 2011 to June 2012), and recruited 959 pregnant women. Patient recruitment was consecutive. The HBsAg was tested using the Monalisa HBsAg Ultra ELISA kit. Other hepatitis B markers were equally tested.

We used the statistical package for social sciences (SPSS) version 14.0 software to conduct a quantitative analysis of the derived data. Simple descriptive statistics such as means, standard deviations, and proportions were used to describe the data. We tested for association in categorical variables using the chi-squared (χ2) test. The odds ratio (OR) and the corresponding 95% confidence intervals (95% CI) were used to summarise the strength of association between specific binary exposure and outcome variables. The level of statistical significance for the study was set at p < 0.05.

**Results:**

The prevalence of hepatitis B infection (HBsAg) among antenatal clinic attenders in our setting was 7.7%. Amongst these women, just 5.4% were previously aware of their HBsAg status. The rate of HBV infectivity was high, with 28% of HBsAg positive women having evidence of HBeAg in their plasma, and up to 45.8% of these women lacking antibodies against hepatitis B e antigen (anti-HBe). About 41% of the pregnant women had had previous contact with HBV as evidenced by the positive status for anti-HBc.

Just 2.7% of the pregnant women had previously been vaccinated against HBV. The mean age for HBsAg positivity in our setting was 26.9 ±4.7 years, and the most affected age group was the 25 – 29 years age group. There was no statistically significant association between age or other socio-demographic risk factors and HBsAg status. Numerous risk factors for HBV acquisition exists in our settings, but amongst these, only a history of a contact with hepatitis B infection was found to be significantly associated with HBsAg positivity (OR 1.63, 95% C.I 1.15-2.30). Finally, the coinfection rate of HBV/HIV was 0.74%.

**Conclusion:**

The prevalence of hepatitis B among pregnant women in Cameroon is high, and the pattern tends towards high infectivity and therefore increased risk of perinatal HBV transmission. These highlight the need to step up preventive efforts against hepatitis B infection and perinatal HBV transmission in our community.

## Background

Infection with Hepatitis B Virus (HBV) remains a serious public health problem worldwide and is a major cause of morbidity and mortality in Africa and Asia [1-3]. Perinatal transmission is one of the commonest modes of HBV transmission worldwide [4]. The Global Advisory Group on the Expanded Program on Immunisation recommended that countries with a more than 2% prevalence of HBV carriers should add hepatitis B vaccine into their routine infant immunization schedules, a recommendation which was endorsed by the World Health Assembly [5]. Consequently the routine screening of pregnant women for hepatitis B surface antigen (HBsAg) is recommended by the World Health Organisation [6].

Chronic Hepatitis B virus infection affects approximately 350 million people worldwide, half of whom acquired the infection from perinatal transmission or in early childhood [4,7]. This perinatal transmission of HBV leads to severe long term sequelae [8]. Children born to mothers who are positive for hepatitis B surface antigen (HBsAg) and Hepatitis B e antigen (HBeAg) have a 70-90% chance of perinatal acquisition of HBV infection, and over 85-90% of them will eventually become chronic carriers of the disease. Chronic carriers of HBV have an increased lifetime risk of dying from hepatocellular carcinoma and liver cirrhosis (25% risk) [9,10], and remain the main reservoir for continued transmission of HBV [11]. Many of them eventually become mothers themselves, thus perpetuating the cycle [12].

Cameroon is a high endemic area for HBV infection with a prevalence rate > 8% This implies perinatal and early childhood transmission are therefore the main means of HBV infection in Cameroon [13,14], with the potential risk of chronic HBV infection. Yet the response of Cameroon to the call of universal screening of pregnant women for HBV has been timid. The screening of antenatal clinic attenders for HBV is not yet a routine practice in most health settings in Cameroon. Moreover the implementation of routine vaccination of newborns against hepatitis was only adopted in 2005.

Few studies have however evaluated the prevalence of HBV in the general Cameroonian population or among antenatal attendants [15-18]. Amongst these, Ndumbe et al reported a prevalence of 9.6% in the general Cameroonian population [16], while Chiaramonte et al reported an alarming 19.9% prevalence among school children in an urban setting in Cameroon [15]. Working on antenatal clinic attendants, Ndumbe et al reported a prevalence rate of HBV of 5.4% among rural pregnant women in Cameroon [17], while Kfutwah et al recently reported a prevalence of 7.85% among urban pregnant women [18]. The study by Kfutwah et al is the lone study carried out in an urban setting in Cameroon. However, their results are based on evaluation of blood samples collected from antenatal clinic attenders in the year 2000 during a pilot program for the prevention of mother-to-child transmission of the Human Immuno-deficiency virus (HIV). Consequently, their results are highly informative of the state of the epidemiology as it was some 12 years ago, but probably do not reflect the state of the epidemic in Cameroon today.

Given that HBV leads to serious sequelae, it is important that its epidemiology should continuously be examined [19]. It is therefore timely for current studies to be performed to better characterise the epidemiology of HBV among pregnant women in Cameroon and to assess whether there has been a shift in the epidemiology of this condition over time. This will help inform the Cameroonian Ministry of health on the state of the art of this epidemic, and whether or not there is a need to prioritise this problem.

The aim of this study was to determine the prevalence, correlates and patterns of Hepatitis B surface antigen among pregnant women attending antenatal care in Yaounde-Cameroon. We carried the research a step further by assessing the infectivity of these women to shed more light on the possible risks of perinatal HBV transmission.

## Methods

This was a cross-sectional study conducted at the Yaounde University Teaching Hospital (CHUY), the Biyem-Assi (BADH) and the Cite Verte District Hospitals (CVDH) of Yaounde, the capital of Cameroon. The University Teaching Hospital is a tertiary hospital in the Yaounde, receiving a wide variety of patients not only from other hospitals in Yaounde itself, but also from other hospitals nationwide. The Cite Verte and Biyem-Assi District Hospitals are secondary care facilities within Yaounde, catering for a wide variety of patients as well, both rural and urban. By selecting patients from these three facilities we believe our sample was largely representative of all categories of pregnant women in Yaounde.

Ethical approval to conduct this study was obtained from the Ethical committee of the International Research Centre on HIV/AIDS of the Chantal Biya Foundation. Each patient provided written informed consent to participate in the study.

The study recruited 959 pregnant women attending antenatal care in one of the above cited hospitals during the study period. The sample size was calculated using the standard formula for sample size calculation N = z^2^pq/d^2^ (where z = the standard normal deviation at 1.96 (which corresponds to a 95% confidence interval), p = the prevalence of Hepatitis B in the general Cameroonian population, estimated at 10%; q = 1 – p; and d = the degree of precision expected = 0.05).

The study lasted 15 months, from March 2011 to June 2012 and we employed a consecutive sampling for data collection, requesting consent from all antenatal clinic attenders in the selected health facilities during the study period. We included women with a confirmed pregnancy who provided written informed consent to participate in the study. Pregnancy was confirmed by any of the following means: positive pregnancy test (serum or urine B-HCG); ultrasound; quickening; and the presence of fetal heart tones by either auscultation or Doppler.

We excluded all women who refused to give consent, and those who presented very early with a doubtful pregnancy. We equally excluded women with gestational trophoblastic disease/molar pregnancy, ectopic pregnancy and complete/incomplete/missed abortions.

Following informed consent, all participants were interviewed using an interviewer administered pretested questionnaire. There were no personal identifiers on the questionnaires. This questionnaire was applied to gather relevant socio-demographic data of the women as well as predisposing factors for hepatitis B infection obtained following a literature review. These included a previous history of spontaneous abortion or still birth, history of blood transfusion, a history of previous surgery, history of previous jaundice, a history of scarifications/tattoos, having a contact with a known history of hepatitis B infection or jaundice (used as a proxy indicator for hepatitis B infection) and a previous history of sexually transmitted infection (used as a proxy indicator for multiple sexual partners). We collected information on these risk factors to subsequently test their association with HBV infection among pregnant women in our community.

After completion of the questionnaire, 5 ml of blood was aseptically collected from the brachial/antebrachial vein using a disposable syringe into Ethylene Di-amine Tetra-acetic Acid (EDTA) vials, in order to prevent clotting. These vials were properly labeled with the patients’ name and date of collection. The blood was either submitted directly to the laboratory, or temporarily stored in a refrigerator overnight and later transferred the following day in cooling packs to the ‘Chantal Biya’ International Reference Centre for Research on HIV/AIDS Prevention and Management (CIRCB) Yaounde for biological analysis.

At the laboratory, the blood was them brought out of the cooling packs and allowed to equilibrate with room temperature. Plasma was them separated for HBV assay by centrifugation at 6.000 revolutions per minute for 5 minutes. After centrifugation, the plasma was tested for HBsAg using the Monalisa HBsAg Ultra ELISA kit (BIO-RAD Laboratories). Positive and negative plasma controls were run alongside each test. Most samples were equally evaluated for the Hepatitis B core antibody (anti-HBc) in order to determine previous exposure to HBV within the study population. After identification of HBsAg positive samples, and given the limited availability of testing kits, a random sample of plasma amongst these was chosen for evaluation of infectivity by testing for the Hepatitis B e antigen (HBeAg). For the same reason as above, not all the HBsAg positive samples were tested for anti-HBe.

Rapid tests for HIV types 1 and 2 were equally run on the samples using the DETERMINE HIV ½ test kit. Seven of the women who consented to the study choose to specifically opt out of the HIV testing, which is a routine test recommended for all pregnant women in Cameroon. They were not included in the specific analysis dealing with HIV and HBV.

Results were made available to the patients before delivery. All women who tested positive for HBsAg were counseled on their status, the modes of disease transmission, the need for neonatal immunization and close-contact screening against hepatitis. They were then referred for further evaluation and management by a gastroenterologist in the Yaounde University Teaching Hospital. Provisions were equally made for immunoprophylaxis and vaccination against Hepatitis B for the newborns at birth.

We used the statistical package for social sciences (SPSS) version 14.0 software to conduct a quantitative analysis of the derived. The variables were summarised and examined. Simple descriptive statistics such as means, standard deviations and proportions were used to describe the data as appropriate.

Where indicated by the research question, variables were cross-tabulated and hypotheses tested by applying appropriate statistical tests. We tested for association in categorical variables using the chi-squared (χ2) test, reporting corresponding p-values. In case of small numbers in a given group (<5), the Fischer’s exact test was used instead, and the corresponding p-value reported. Wherever possible, analyses were done using the ungrouped variables to retain optimum information content/power. For the purpose of hypothesis testing, a control group selected from the pregnant women who were HBsAg negative, and matched, for age, educational level, marital and professional status with the cases, was used to compare the strength of the association between reported risk factors and infection with Hepatitis B virus. We opted for this nested case–control design within our cross section as it is a validated approach and may actually be advantageous over a full cross sectional design [20]. The odds ratio and the corresponding 95% confidence intervals (95% CI) were used to summarise the strength of association between specific binary exposure and outcome variables. The level of statistical significance for the study was set at p < 0.05.

The primary outcome measure was the detection of the presence of Hepatitis B surface antigen (HBsAg) in the plasma of pregnant women, and their association with known risk factors for maternal infection. Secondary outcome measures included the assessment of markers of hepatitis B infectivity (hepatitis B ‘e’ antigen (HBeAg), antibody against the Hepatitis B ‘e’ antigen) among HBsAg positive participants, and coinfection with the Human Immuno-deficiency Virus (HIV).

## Results

**Figure 1:**
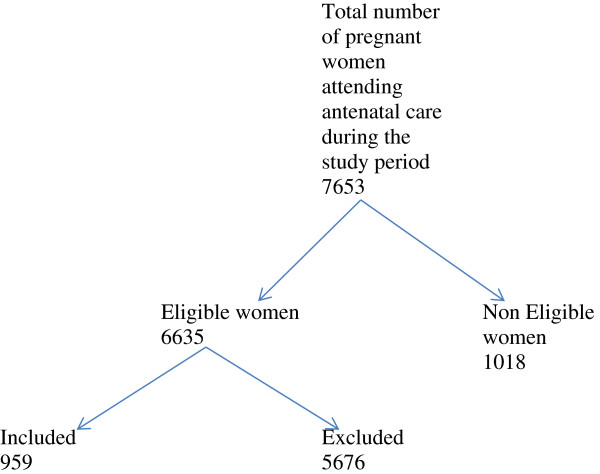
Sketch outlining the selection of the sample population.

Figure 1 illustrates all the pregnant women attending antennal care during the study period and the sample eventually recruited into the study.

Among the 959 patients who participated in this study, the ages varied between 15 and 43 years with a mean age of 27.6 ±5.2 years. The mean gestational age of our patients was 29.2 weeks; (minimum = 6 weeks; maximum = 42 weeks). Most of these women (97.3%) had never been vaccinated against Hepatitis B. Table 1 summarises the socio-demographic characteristics of the study participants.

**Table 1:** Socio-demographic characteristics of study participants

**Characteristic**	**Number (n = 959)**	**Percentage (%)**
**Age (Years) (Mean = 27.6; SD = 5.2)**		
≤ 15	3	0.3
16 – 20	50	5.2
21 – 24	224	23.4
25 – 29	356	37.1
30 – 34	198	20.6
35 – 39	114	11.9
40 +	14	1.5
**Marital status**		
Single	181	18.9
Cohabiting	186	19.4
Married	592	61.7
**Educational level**		
Uneducated	4	0.4
Primary education	101	10.5
Secondary education	537	56.0
University and beyond	317	33.1
**Occupational status**		
No formal employment	505	52.7
Students	232	24.2
Civil servants	157	16.4
Private sector workers	65	6.8
**Gravidity**		
Primagravida	305	31.8
Multigravida	654	62.2

The Hepatitis B surface antigen (HBsAg) was detected in the plasma of 74 of these women, giving an overall HBsAg prevalence of 7.7%. Just 4 of these women (5.4%) were previously aware of their HBsAg status. Among the 74 women who tested positive for HBsAg, results for HBeAg testing was available for 25. Amongst these 25 women, 7 (28%) tested positive for HBeAg, thus indicating that this proportion of patients was highly infectious, and therefore likely to transmit the virus to their offspring. Results for testing for anti-HBe were available for 48 HBsAg (+) patients. Amongst these, 45.8% (n = 22) lacked anti-HBe. This implies a higher proportion of women were thus at risk of active viral replication, than was estimated by our assessment of HBeAg alone. The remainder (54.2%) had anti-HBe, indicating they were seroconverting.

Previous exposure to HBV was evaluated by assessing the prevalence of anti-HBc within the study sample. This marker was evaluated in 644 patients. Of these, 263 (40.8%) were positive for total anti-HBc, hence had serologic evidence of previous HBV exposure.

The mean age among pregnant women who were HBsAg positive was 26.9 years. The prevalence of HBsAg was highest among the 25 – 29 year age group (Table 2). There was no statistically significant association between the socio-demographic characteristics evaluated (age, gravidity, marital status, and educational level), and HBsAg status.

**Table 2:** HBsAg prevalence by age among the pregnant women

**Age group**	**Number (n = 959)**	**HBsAg + (%)**	**HBsAg – (%)**
≤ 15	3	0 (0.0%)	3 (100.0%)
16 – 20	50	5 (10.0%)	45 (90.0%)
21 – 24	224	13 (5.8%)	211 (94.2%)
25 – 29	356	38 (10.7%)	318 (89.3%)
30 – 34	198	14 (7.1%)	184 (92.9%)
35 – 39	114	3 (2.6%)	111 (97.4%)
40+	14	1 (7.1%)	13 (92.9%)

(FE χ ^2^ = 10.4 df = 6 p = 0.11).

As stated in the methods, a control group matched for age and professional status was used to compare the strength of the association between reported risk factors and infection with Hepatitis B virus. This gave a new sample of 124 patients, with 59 cases and 65 controls. In this new sample, 23 HBsAg positive patients (39.0%) reported having a contact with a known history of hepatitis B infection or jaundice (used as a proxy indicator for hepatitis B infection). The association between a history of a contact with hepatitis and HBsAg status was statistically significant, with a significantly higher risk of being HBsAg-positive amongst those with a contact with HBV or jaundice, relative to those without such a contact (OR 1.63, 95% C.I 1.15 – 2.30). The most commonly cited contacts were immediate family members including the mother, the father, brothers and sisters, cousins and aunts. One patient reported that her mother in law was known to be HBV positive. One patient who identified her father as being HBV positive reported he was a worker in a blood bank.

A previous history of spontaneous abortion or still birth, history of blood transfusion, a history of previous surgery, history of previous jaundice, a history of scarifications/tattoos and a previous history of sexually transmitted infection were not found to be statistically significantly associated with the HBsAg status (Table 3).

**Table 3:** Association between HBsAg status and the predisposing factors

**Risk factor**	**Number**	**HBsAg(+)**	**HBsAg(−)**	**OR**	**95% C.I**
**History of abortion/stillbirth**	**124**				
Yes	47	22	25	0.97	0.66 – 1.43
No	77	37	40		
**History of blood transfusion**	124				
Yes	6	2	4	0.69	0.22 – 2.17
No	118	57	61		
**History of surgery**	124				
Yes	16	3	13	0.36	0.13 – 1.02
No	108	56	52		
**History of scarification/tattoo**	124				
Yes	23	9	14	0.79	0.46 – 1.37
No	101	50	51		
**History of jaundice**	124				
Yes	14	8	6	1.23	0.75 – 2.02
No	110	51	59		
**Contact with jaundice/Hepatitis B**	124				
Yes	35	23	12	1.63	1.15 – 2.30
No	89	36	53		
**History of STI**	124				
Yes	19	9	10	0.99	0.59 – 1.66
No	105	50	55		

*STI* Sexually Transmitted Infection, *OR* Odds ratio; 95% C.I = 95% confidence interval.

In our study sample 80 (8.4%) women tested positive for HIV (n = 952). Seven of these 80 women were equally infected with the hepatitis B virus. This implies the coinfection rate HIV/HBV in our study population is 0.74% (7/952).

The relationship between HIV status and HBsAg status was not statistically significant (Table 4).

**Table 4:** HIV status and HBsAg status

	**HIV (+)**	**HIV (−)**	**Total**
**HBsAg (+) (n = 74)**	7 (8.8%)	67 (7.7%)	74
**HBsAg(−) (n = 878)**	73 (91.3%)	805 (92.3%)	878
**Total**	80	872	952

(OR 0.88, 95% C.I 0.42 – 1.84).

## Discussion

The results of our study indicate that the prevalence of HBsAg among antenatal clinic attenders in Yaounde is 7.7%. Cameroon is generally considered an area of hyper-endemicity for hepatitis B infection (prevalence >8%) [14]. This fact is strongly supported by the prevalence of HBsAg among blood donors such as the 10.8% reported by Noah et al [21] 10.7% reported by Mbanya et al [22] and recently the 12.4% reported by Fouelifack et al [23] all among blood donors in Yaounde Cameroon. Our prevalence of HBsAg of 7.7% among pregnant women is comparable to these figures, thus suggesting that seroprevalence of HBsAg among pregnant women may be used as a proxy measure of this condition in the general population.

After Kfutwah et al [18] this is the second study on hepatitis B carried out among pregnant women in an urban setting in Cameroon. Our HBsAg seroprevalence is identical to the prevalence of 7.85% among pregnant women reported by Kfutwah et al [18] earlier on this year in Yaounde, despite working on blood samples collected from pregnant women more than 10 years ago. Our results are however higher than the earlier reports of HBsAg prevalence among pregnant women in Cameroon by Ndumbe et al who reported a 5.4% prevalence among pregnant women in a rural setting [17]. One obvious difference between our study and that of Ndumbe et al is the different settings and study populations. One may estimate that urban dwellers probably have a higher risk of acquiring the infection that those in rural settings. This hypothesis however needs to be evaluated.

Our prevalence of HBsAg among pregnant women of 7.7% is comparable to the 8.3% HBsAg prevalence among pregnant women reported by Luka et al [24] in an Urban setting in Nigeria, and by Eke et al [19] still among pregnant women in a rural setting still in Nigeria. They are comparable with the 6.4% HBsAg prevalence reported in Ghana [25], the 6.5% HBsAg prevalence reported in Congo [26], the 9.3% HBsAg prevalence reported in Kenya [27] and the 10.7% HBsAg prevalence in Burkina Faso [28], all among pregnant women. Our findings are significantly lower than the 25% prevalence of HBsAg reported by Madzime et al among pregnant women in Zimbabwe [29]. On the contrary, our results are higher than the 1.2% HBsAg prevalence reported among antenatal clinic attenders in South Africa [30].

A high proportion of our patients showed evidence of previous exposure to HBV as evidenced by the 40.8% of anti-HBc prevalence within our study population. This proportion is lower than the 85% anti-HBc seroprevalence reported by Ndumbe et al among pregnant women in a rural setting in Cameroon. It is however higher than the anti-HBc seroprevalence among pregnant women in the developed world, with reports ranging from 7.1% in Switzerland [31], through 13.4% in France [32], and 29.65% anti-HBc seroprevalence among pregnant women in China [33].

Our study equally indicates that just 2.7% of pregnant women have ever been vaccinated against HBV. This shows a massive lack of awareness concerning HBV infection in the general Cameroonian population and the need for anti HBV vaccination. It further indirectly highlights the present inadequacy of community sensitization activities by our health authorities on sensitizing the Cameroonian population about HBV infection, and the importance of anti HBV vaccination.

These two preceding facts combined highlight the fact that HBV is very much present in our community. They further indicate that even though Cameroon is a hyper-endemic area for HBV, where transmission is generally considered to be vertical, horizontal transmission is also an important mode of HBV acquisition as presumed by Kfutwah et al [18]. Given that very few women have ever been vaccinated against HBV, the risk of HBV acquisition in our community is high. We however did not find any reports that had looked into the vaccination status against HBV of pregnant women within our community. Our study therefore strongly highlights this important fact which hitherto was apparently neglected by HBV researchers in Cameroon.

The hepatitis e status, and the HBV viral load are both factors known to be associated with vertical HBV transmission [18]. We did not evaluate vertical transmission in our study, but used the presence of HBeAg which is a marker of high infectivity, as a proxy measure for the risk of vertical transmission of HBV. HBeAg positive patients are known to have a high viral load, and transmit HBV to their offspring [18]. We found that 28% of HBsAg positive patients in our sample were equally HBeAg positive. This was largely in contrast with Kfutwah et al who found no HBeAg positive samples amongst patients who tested positive for HBsAg. It is known that the risk of vertical transmission and resulting chronic carrier infection from an HBsAg (+) mother to her baby is approximately 90% in HBeAg positive pregnant women with high HBV DNA titres [34,35]. Hence our results do not support the assertion by Kfutwah et al who stated that ‘this form of transmission (vertical transmission) could play a negligible role in HBV transmission in Cameroon’ [18]. Our findings suggest that vertical transmission possibly is a very important means of HBV transmission in Cameroon.

Reports from sub-Saharan Africa indicates that sero-conversion of anti-HBe occurs before the age of 15 to 16 years, with the consequence that most reproductive age women do carry anti-HBe [36]. Our findings indicated that up to 45.8% of HBsAg positive patients lacked this antibody. This was an indirect indicator of the infectivity of these women. In addition, HBV chronicity is associated with early infection (fetal, perinatal, neonatal or early infancy) [17]. The rate of HBV positivity among pregnant patients in our settings is quite high, suggesting that early infection plays an important role. These facts further support our belief that vertical transmission is indeed a very important means of HBV transmission in Cameroon.

In our study, the mean age of HBsAg positivity was 26.9 years, and HBsAg was highest among the 25 – 29 years age group (Table 2). This HBsAg seroprevalence was equally high (10%) among the 16 – 20 years age group (Table 2). Our findings are in accordance with Vazquez-Martinez et al who reported that the average age of women infected with hepatitis B virus was 26 years [37]. Our findings somewhat tally with Ndumbe et al in Cameroon [17], and Eke et al in Nigeria [19] who found the highest prevalence of HBsAg among the 10 – 19 age group (10.4%), and the 15 – 19 age group (16.7%) respectively. A possible reason for the slightly higher HBsAg prevalence in the 25 – 29 years age group is the fact that between these ages, many women in urban areas of Cameroon are likely to get married and become pregnant. They are therefore likely to present for the first time in antenatal care, and can thus be easily picked up by screening.

Other socio-demographic characteristics were evaluated to determine whether these were associated with a risk of HBV acquisition. This included the marital status of the patients, their educational level, their professions, and their gravidity. Despite reports from other studies that some of these factors may be linked with the risk of HBV acquisition, we did not find a statistically significant association between any of these factors and the risk of HBV infection among our study subjects.

Despite the high prevalence of HBsAg among pregnant women in our settings, just one predisposing factor for HBV infection showed statistical significance. This result was in accordance with Summer et al who showed that most of those testing positive for HBsAg had just one or two predisposing factors for hepatitis B surface antigen [38]. It was noted from this study that previous contact with someone with hepatitis B infection was a statistically significant predisposing factor for HBsAg infection. Our findings are in accordance with other reports which indicate that the risk of HBV transmission is high in people who are in contact chronically infected HBV subjects [19,39-41].

There was no significant association between the other risk factors and HBsAg positivity. This is in accordance with studies carried out in neighboring Nigeria [19,42]. We could not make any direct comparisons with previous reports in Cameroon as none of these evaluated the significance of supposed predisposing factors for HBV among pregnant women. To the best of our knowledge therefore, our study is the first study evaluating the relative importance risk factors for HBV acquisition among pregnant women in Cameroon.

The association between HIV and HBV was not statistically significant in our study. Ahmed et al in Malawi equally failed to find any statistical evidence for the association between HIV positivity and HBV infection [43]. The HIV/HBV coinfection rate in our study population was 0.74%. Our results are comparable to the 0.7% HIV/HBV coinfection rate among pregnant women in Awka, Nigeria [44], but are lower than the 4.2% HIV/HBV coinfection rate among pregnant women reported by Eke et al [19] still in Nigeria. Reports from other African settings equally indicate that HIV/HBV coinfection rate among pregnant women is low. Pertinent amongst these, are reports from Burkina Faso showing a co-infection rate of 0.88% [45], and another in Benin City, Nigeria which showed a low coinfection rate of 0.20% [46]. Our findings are in contrast with those of Ndumbe et al in Cameroon, who did not find any coinfection with HBV among the 13 pregnant women found to be HIV positive [17]. One possible reason was that the number of HIV positive cases in the study by Ndumbe et al was relatively small, and the HIV epidemic was probably still in its early stages in Cameroon at the time.

In our survey, the HBsAg positivity rate was similar in HIV-positive (8.8%) and HIV-negative pregnant women (7.7%). This finding was similar to that reported by Kfutwah et al who found an HBsAg positivity rate of 9% and 7% among HIV-positive and HIV-negative pregnant women respectively [18]. Similar findings have equally been reported in Cote d’Ivoire [47]. The role of sexual transmission of HBV can therefore not be neglected among this population of pregnant women. The fact that HIV and some hepatitis viruses share common modes of transmission has often raised questions concerning the potential of the hepatitis viruses to increase the virulence of HIV infection, and/or influence the progression of symptomatic HIV infection in asymptomatic patients [48,49]. The low coinfection rate of HIV/HBV in our study population is a rather consolatory finding given that the prevalence of both of these infections is high among pregnant women in Cameroon.

The implications of our research findings are numerous. The 7.7% prevalence of HBsAg among pregnant women in our community is an indication that pregnant women serve as an important reservoir to fuel the HBV infection in the general Cameroonian population. Furthermore, the fact that over 28% of HBsAg positive pregnant women were also positive for HBeAg implies the risk of perinatal and childhood transmission of HBV is very high within our community. Efforts are therefore needed to contain this situation. A way forward could be to strengthen the policy on routine HBV testing in antenatal care. This had previously been suggested [18]. The inclusion of HBsAg as a routine prenatal test should be accompanied by appropriate facilities for management of positive cases. This screening would also enable the timely passive and active immunization of newborns born to HBsAg positive mothers in order to minimize if not avert seroconversion (57). Other preventive measures would include the provision of pre and post-test counselling services for HBV on the same day, the availability of rapid HBV-testing facilities within the clinic and increased confidentiality at every level of the program. There should equally be provision for active/passive immunisation for the infants of infected mothers. Screening of pregnant women will also enable the screening of close contacts of those infected, and these individuals can then either be vaccinated or managed as appropriate.

The similarity between our HBsAg seroprevalence and pregnant women and that reported by Kfutwah et al [18] implies there has not been any significant change in the epidemiology of this condition within the past 10 years in Cameroon. It therefore highlights the fact that preventive efforts against horizontal HBV transmission have had little or no impact within our community for the time being. This is further supported by the low vaccination rates against HBV despite the existence of a safe and effective vaccine. In addition, contact with a chronically infected subject was the lone risk factor significantly associated with HBV infection within our community. These call for a need to step up preventive efforts against horizontal HBV transmission within our community if we hope to halt and subsequently reverse this epidemic. Possible measures could include universal vaccination against HBV of at risk groups such as heal workers, blood bank workers, and those with a contact known to be infected with HBV.

Based on this study, we propose the following recommendations: prenatal screening for HBV should be included as part of the routine tests for pregnant women in our community; prenatal counseling on HBV should be instituted within our community as this could help to raise the awareness of HBV among future mothers and might be linked to higher follow up rates in HBV immunization independent of the mothers’ HBV status; anti hepatitis B vaccination for all new borns should be reinforced, this irrespective of the mother’s HBV status; provisions should be made for appropriate follow-up and subsequent management of HBV positive pregnant women identified through screening, during and after their pregnancy; Hepatitis B immunoglobulin should be made available to enable passive immunisation of infants from HBV infected mothers; there should be increased collaboration between maternity and vaccination services to enable timely vaccination of infants of HBV infected mothers.

### Strenghts and weaknesses of our study

The major strength of our study lies in the methodology and the large sample size. Patients were recruited prospectively and as such, our results demonstrate the state of the art of the epidemic as it presently is. In addition, it was a multicenter study, capturing a diverse population of pregnant women (both rural and urban) within and around Yaounde which is the capital city of Cameroon, hence truly reflects the epidemiology of HBV within our community.

A further strength lies in the fact that certain practical challenges were identified. In the course of this research it became obvious that hepatitis B immunoglobulin for passive immunisation of the newborns of HBV infected mothers is not commonly available in our settings. Even the provision of HBV vaccine within 12 hours of delivery was rather challenging. Our study also highlighted worries concerning breastfeeding in HBV infected mothers. This permitted the education of patients and health personnel alike as they were informed that there is no evidence that breastfeeding from an HBV infected mother poses an additional risk of HBV infection to her infant, even in the absence of immunisation [50].

Our data was limited by the fact data on reported risk factors depended on the memories of study participants, and there was no other means to verify the truthfulness of this information. Moreover, it was noticed that the probing into these risk factors created an atmosphere of suspicion of HBV infection among study participants, and may have biased some of the responses. Also, the rate of HBeAg was measured in one third of the HBsAg positive women and anti-HBe was measured in two thirds of HBsAg-positive women all due to financial constraints. Despite the fact that these samples were random hence probably representative samples, considering all the samples would have been the ideal.

## Conclusion

The prevalence of hepatitis B among pregnant women in Cameroon is high and the pattern tends towards high infectivity and therefore increased perinatal transmission. The epidemiology of this condition has been stagnant over the past 10 years, and indicates that pregnant women serve as a source of the infection in the general population as well. These facts highlight the need for preventive efforts against hepatitis B infection in pregnant women in Cameroon.

## Competing interests

The authors declare that they have no competing interests.

## Authors’ contributions

FM and JF conceived the research idea. FM, JT and WT did the patient recruitment and follow. FM made the first draft. PN and MK proof-read the paper and made vital corrections. All authors reviewed several versions of the manuscript. All authors read and approved the final manuscript.

## Pre-publication history

The pre-publication history for this paper can be accessed here:

http://www.biomedcentral.com/1471-2393/13/158/prepub
